# Virtual monoenergetic images from spectral detector computed tomography facilitate washout assessment in arterially hyper-enhancing liver lesions

**DOI:** 10.1007/s00330-020-07379-3

**Published:** 2020-11-12

**Authors:** R. P. Reimer, N. Große Hokamp, A. Fehrmann Efferoth, A. Krauskopf, D. Zopfs, J. R. Kröger, T. Persigehl, D. Maintz, A. C. Bunck

**Affiliations:** 1 Department of Diagnostic and Interventional Radiology, Faculty of Medicine and University Hospital Cologne, University of Cologne, Kerpener Str. 62, 50937 Cologne, Germany; 2 Department of Diagnostic and Interventional Radiology, University Düsseldorf, Medical Faculty, 40225 Düsseldorf, Germany

**Keywords:** Carcinoma, hepatocellular, Tomography, X-ray computed, Diagnostic imaging

## Abstract

**Objectives:**

To investigate whether the increased soft tissue contrast of virtual monoenergetic images (VMIs) obtained from a spectral detector computed tomography (SDCT) system improves washout assessment of arterially hyper-enhancing liver lesions.

**Methods:**

Fifty-nine arterially hyper-enhancing lesions in 31 patients (age 65 ± 9 years, M/W 20/11) were included in this IRB-approved study. All patients underwent multi-phase SDCT for HCC screening. MRI, CEUS or biopsy within 3 months served as standard of reference to classify lesions as LiRADS 3 or 4/5. VMIs and conventional images (CIs) were reconstructed. Visual analysis was performed on 40, 60, and 80 kiloelectronvolt (keV) and CIs by 3 radiologists. Presence and visibility of washout were assessed; image quality and confidence of washout evaluation were evaluated on 5-point Likert scales. Signal-to-noise ratio (SNR), lesion-to-liver contrast-to-noise ratio (CNR) (|HU_lesion_–HU_liver_|/SD_liver_) and washout (|HU_lesion_–HU_liver_|) were calculated. Statistical assessment was performed using ANOVA and Wilcoxon test.

**Results:**

On subjective lesion analysis, the highest level of diagnostic confidence and highest sensitivity for the detection of lesion washout were found for 40-keV VMIs (40 keV vs. CI, 81.3 vs. 71.3%). Image quality parameters were significantly better in low-kiloelectronvolt VMIs than in CIs (*p* < 0.05; e.g. SNR_liver_: 40 keV vs. CIs, 12.5 ± 4.1 vs. 5.6 ± 1.6). In LiRADS 4/5 lesions, CNR and quantitative washout values were significantly higher in 40-keV VMIs compared to CIs (*p* < 0.05; e.g. CNR and washout in 40 keV vs. CIs, 2.3 ± 1.6 vs. 0.8 ± 0.5 and 29.0 ± 19.1 vs. 12.9 ± 6.9 HU, respectively).

**Conclusion:**

By increasing lesion contrast, low-kiloelectronvolt VMIs obtained from SDCT improve washout assessment of hyper-enhancing liver lesions with respect to washout visibility and diagnostic confidence.

**Key Points:**

*• Low-kiloelectronvolt virtual monoenergetic images from spectral detector CT facilitate washout assessment in arterially hyper-enhancing liver lesions.*

*• Image quality and quantitative washout parameters as well as subjective washout visibility and diagnostic confidence benefit from low-kiloelectronvolt virtual monoenergetic images.*

**Electronic supplementary material:**

The online version of this article (10.1007/s00330-020-07379-3) contains supplementary material, which is available to authorized users.

## Introduction

Hepatocellular carcinoma (HCC) may be diagnosed by contrast-enhanced (CE) magnetic resonance imaging (MRI), computed tomography (CT), ultrasound (CEUS) or a combination of these modalities without the need for a histological confirmation by biopsy [1, 2]. Many studies and meta-analyses investigated and compared the diagnostic performances of the different modalities with the predominant result that MRI provides the highest accuracy, especially in small liver lesions [2–4]. Although, these studies slightly favour MRI over CT, both are equally recommended as modality of choice for the diagnostic evaluation of patients at risk of HCC, due to their high sensitivity and coverage allowing a comprehensive assessment of the entire liver [1, 2]. However, MRI may be contraindicated in some patients or may result in insufficient image quality, e.g. in patients with ascites or in patients incapable of holding their breath. Furthermore, diagnosis may require a confirmation by a second imaging modality if imaging features are equivocal or a lesion has been assigned an intermediate probability for malignancy [2, 5].

Typical radiological findings in HCC include a combination of an arterial hyper-enhancement (APHE) with nonperipheral washout in portal-venous and/or delayed phase in CT/MRI or late washout (> 60s) in CEUS, enhancing capsule and/or lesion threshold growth on a follow-up scan [2, 5, 6]. The assessment of washout, defined as a hypointensity, hypo-density or hypo-echogenicity compared to the adjacent liver parenchyma, can be difficult in cases with only subtle differences [7].

In the past decade, dual-energy CT (DECT) raised interest in the field of liver imaging, since it has been shown to improve soft tissue contrast by means of virtual monoenergetic images (VMIs). DECT systems register low- and high-energy data attenuation profiles enabling a reconstruction of VMIs, which approximate images from an acquisition with a true monoenergetic X-ray beam [8–10].

While different emission-based DECT systems have been available for more than a decade, recently, a detector-based approach was introduced, referred to as spectral detector CT (SDCT). By using a dual-layer detector, it enables a simultaneous detection of low- and high-energy photons with complete temporal and spatial registration, allowing for a retrospective reconstruction of VMIs in a range from 40 to 200 kiloelectronvolt (keV) for every scan [10–12].

VMIs at low kiloelectronvolts are known to improve soft tissue contrast due to the energy dependence of the linear attenuation coefficients while maintaining low image noise throughout the available keV range [10]. Furthermore, multiple studies reported an increase in detectability and conspicuity of arterially hyper-enhancing liver lesions in VMIs using low-kiloelectronvolt values [10, 13–15]. Additional studies showed better visualization of hypodense liver lesions, while only few focused on HCC [14–18].

The aim of our study was to analyse whether the increased soft tissue contrast by means of VMIs improves the washout assessment of arterially hyper-enhancing liver lesions in contrast-enhanced SDCT.

## Material and methods

### Study population

This retrospective study was conducted in compliance with the protocol and the principles laid down in the Declaration of Helsinki, in accordance with the ICH Harmonized Tripartite Guideline for Good Clinical Practice. The institutional review board waived informed consent. A structured search in the radiology information system was performed with the following inclusion criteria: (1) older than 18 years of age, (2) multi-phase contrast-enhanced SDCT for HCC workup between May 2016 and November 2018, (3) examination with a standardized imaging protocol as described below, (4) arterially hyper-enhancing liver lesions of 1 cm or greater and (5) MRI, CEUS or biopsy within 3 months. A maximum of 5 lesions per patient was included.

Exclusion criteria comprised (1) prior locoregional therapy and (2) modified imaging protocol and incomplete image reconstructions. Imaging studies were reviewed using a clinical DICOM-Viewer (Impax EE R20, Dedalus Group). Contrast-enhanced liver MRI, CEUS or biopsy within 3 months served as reference standard to categorize lesions according to the current LiRADS classification system [5, 6]. All biopsy-positive lesions were rated as LiRADs 5 lesions. In total, 31 patients were identified exhibiting a total of 59 arterially hyper-enhancing focal liver lesions (ESM1).

### Acquisition parameters

All CT scans were performed for clinical indications on the same spectral detector CT scanner (IQon Spectral CT, Philips Healthcare). Patients were scanned in cranio-caudal direction and a head-first, supine position. Our institution’s HCC workup protocol for CT examinations contains an unenhanced, arterial, portal-venous and delayed scan of the liver, while the portal-venous scan covers the abdomen and pelvis, with or without a chest scan. The arterial, portal-venous and delayed phases were acquired using an automated bolus-tracking technique (threshold of 150 HU in the abdominal aorta) with a delay of 15, 50 and 240 s, respectively. Administration of 100 ml non-ionic, iodinated contrast media bolus (Accupaque 350 mg/ml, GE Healthcare) followed by a 30-ml saline chaser is routinely performed with an automated injection system at a flow rate of 4.0 ml/s (MEDRAD® Stellant, Bayer Vital GmbH). Tube current modulation was activated in all patients (DoseRight 3D-DOM, Philips Healthcare). Scan parameters were as follows: collimation 64 × 0.625 mm, tube voltage 120 kVp, pitch 0.485. In portal-venous scans covering the chest and abdomen, pitch was 0.671 (Table 1).

**Table 1:** Radiation dose

CT phase	Rotation time (s)	Tube current-time product (mAs)	DLP (mGy × cm)	CTDI_vol_ (mGy)
Unenhanced	0.33	143 ± 46.7	342.1 ± 130.0	13.0 ± 4.2
Arterial	0.5	131.5 ± 50.6	307.9 ± 136.6	12.0 ± 4.6
Portal-venous	0.33	144.1 ± 52.0	871.1 ± 335.7	13.1 ± 4.7
Delayed	0.33	131.8 ± 50.7	309.2 ± 137.3	12.0 ± 4.6

*DLP*, dose-length product; *CTDI*_*vol*_, volumetric CT dose index. Results are means ± standard deviation

### Image reconstruction

Image reconstruction for further analysis was limited to portal-venous and delayed phases only. Conventional images (CIs) were reconstructed using a hybrid iterative reconstruction algorithm (iDose^4^, Philips Healthcare) with a standard soft tissue kernel (B). VMIs of 40–200 keV with a 10-keV increment were reconstructed using a dedicated, hybrid iterative spectral reconstruction algorithm (Spectral, Kernel B, Philips Healthcare). In SDCT, information from each layer is used to generate photoelectric-like and Compton-like images, which are then linearly blended to generate VMIs. Although the detailed reconstruction process remains undisclosed by the vendor, it appears that some sort of noise reduction is used [19]. Denoising for both was set to a medium level (level 3 of 7). All images were reconstructed with a slice thickness of 2 mm and a section increment of 1 mm.

### Qualitative analysis

All 59 included lesions were qualitatively analysed by one board-certified body radiologist (13 years of experience in liver imaging) and two diagnostic radiology residents with 1-year experience in body CT. Analysis was divided into two parts with an interval of 3 months in between to avoid recall bias. For the analysis, reading sessions were prepared by one of the study team members not involved in the blinded reading. Qualitative assessment was limited to CIs and three representative VMI energy levels (40, 60 and 80 keV) of portal-venous and delayed phases, while arterial phase was only used to indicate lesions to be evaluated. The three readers were free to adjust window settings and scroll through the lesions.

In the first part of our analysis, prepared reading sessions displayed the arterial phase with the lesion of interest encircled on the left screen and the portal-venous and delayed phases with the same image reconstruction on the right screen. First, presence of washout was rated (yes/no) for portal-venous and delayed phases, respectively. Second, if washout was present in both phases, readers were asked to decide which phase displayed washout better. Furthermore, overall image quality and diagnostic confidence of washout evaluation were rated on 5-point Likert scales, being adapted from previous studies [14, 17, 20]: for overall image quality, ranging from 1, non-diagnostic, to 5, extraordinary; and for diagnostic confidence of washout evaluation, ranging from 1, none, to 5, extraordinary (ESM2).

As washout was only or better seen on delayed-phase images in 72.2% in the first part of the qualitative reading, the second part of the qualitative analysis as well as the quantitative analysis was limited to the delayed phase only. For the second part of the qualitative analysis, prepared reading sessions displayed the arterial phase with the arterially hyper-enhancing lesion of interest encircled on the left screen and all blinded delayed-phase image reconstructions (40, 60, 80 keV and CIs) in a randomized order next to each other on the right screen. In this reading, readers were asked to choose the image reconstruction with the best washout to liver contrast, hereafter referred to as best washout visibility.

For statistical analysis, washout was only considered present, if confidence was rated moderate or higher, since otherwise the diagnosis probably would have not been made in clinical routine.

### Quantitative analysis

Quantitative analysis was performed using region-of-interest (ROI)-based measurements of mean HU and standard deviation (SD). Circular ROIs were placed in the following structures: 2 in lesions, 2 in adjacent liver parenchyma, 1 in autochthonous back musculature and 1 in portal vein. All ROIs were drawn as large as possible (at least 0.5 cm^2^) and were only adjusted to exclude unrepresentative structures such as vessels, bile ducts, areas of focal liver cirrhosis and fasciae. In heterogeneous lesions, ROIs were placed in areas that visually appeared most hypodense. ROIs were placed on CIs and copied to identical positions in all reconstructed VMIs. Mean and SD of HU were averaged where applicable. Image noise was represented by SD in back musculature. Signal-to-noise ratio (SNR_*x*_) of a ROI_x_ was defined as SNR_*x*_ = HU_*x*_/SD_*x*_, lesion-to-liver contrast-to-noise ratio (CNR) as CNR = |HU_lesion_–HU_liver_|/SD_liver_ being adapted from previous studies [10, 14] and quantitative washout values as |HU_lesion_–HU_liver_|.

### Statistical analysis

All analyses were performed using JMP Software (Version 14, SAS Institute GmbH) unless specified below. To compare groups, we used ANOVA or Wilcoxon tests, adjusted for multiple comparisons if appropriate. A *p* value < 0.05 was considered significant. Continuous variables are reported as mean ± SD and Likert scores as median (quartiles). Sensitivities and specificities are given with corresponding 95% confidence intervals. Inter-rater reliability was determined by means of intra-class correlation estimates (ICCs) using RStudio (Version 1.1.456; RStudio) based on a mean of 3 raters, consistency and 2-way mixed-effects model for the qualitative analysis [21]. Inter-rater agreement was evaluated as described earlier [22].

## Results

### Study cohort

The mean age of patients was 65 ± 9 years; of these patients, 12 (35.5%) were women and 20 (64.5%) men (ESM1). In total, 59 arterially hyper-enhancing lesions were assessed. According to the reference standards, lesions were classified as LiRADS 3 (*n* = 9), 4 (*n* = 2) and 5 (*n* = 48) (Table 2). Mean lesion size was 3.0 ± 2.4 cm (range, 1.0–13.0 cm).

**Table 2:** LiRADS classification according to the reference standard

	LiRADS 3	LiRADS 4	LiRADS 5
MRI, no washout	8 (13.6)	–	–
MRI, no APHE; washout (< 2 cm)	1 (1.7)		
MRI, capsule	–	2 (3.4)	–
MRI, washout	–	–	16 (27.1)
CEUS, washout	–	–	3 (5.1)
Biopsy, HCC	–	–	21 (35.6)
MRI and CEUS, washout	–	–	2 (3.4)
MRI, no washout; CEUS, washout	–	–	1 (1.7)
MRI, no washout; Biopsy, HCC	–	–	1 (1.7)
MRI, washout; Biopsy, HCC	–	–	3 (5.1)
CEUS, washout; Biopsy, HCC	–	–	1 (1.7)

*LiRADS*, Liver Imaging Reporting and Data System; *MRI*, magnetic resonance imaging; *APHE*, Arterial phase hyper-enhancement; *CEUS*, contrast-enhanced ultrasound; *HCC*, hepatocellular carcinoma. Results are lesion number (%)

### Qualitative analysis

The overall intraclass correlation between the 3 independent readers was 0.897 (0.883–0.909), indicating an excellent interreader reliability. Regarding washout evaluation, the overall ICC was 0.915 (0.895–0.933), in 40 keV 0.857 (0.780–0.910), in 60 keV 0.958 (0.936–0.974), in 80 keV VMIs 0.904 (0.852–0.940) and in CIs 0.936 (0.901–0.960), and regarding confidence of washout evaluation 0.707 (0.635–0.766), 0.789 (0.674–0.867), 0.669 (0.490–0.793), 0.565 (0.330–0.727) and 0.781 (0.662–0.862), respectively.

Overall image quality was rated best in 60-keV VMIs, while 60-keV VMIs and 80-keV VMIs were rated significantly better compared to CIs (both *p* < 0.05); 40-keV VMIs were rated slightly better compared to CIs without reaching statistical significance (*p* > 0.05) (Table 3).

**Table 3:** Results of qualitative analysis

	CIs	40-keV VMIs	60-keV VMIs	80-keV VMIs
Image quality	4 (3–5)	4 (3–5)	4 (4–5)	4 (4–5)
Washout				
Sensitivity, %	71.3 (63.6–78.0)	81.3 (74.3–86.8)	78.7 (71.4–84.5)	74.7 (67.2–81.0)
Specificity, %	96.3 (81.7–99.3)	88.9 (71.9–96.1)	92.6 (76.6–97.9)	92.6 (76.6–97.9)
Confidence	4 (3–5)	5 (4–5)	5 (4–5)	4 (3–5)
Best visible, %	4.4	88.3	5.1	2.2

*CIs*, conventional images; *VMIs*, virtual monoenergetic images. Results are median (quartiles), unless specified

The confidence of washout evaluation was rated in descending order from 40-keV, 60-keV and 80-keV VMIs to CIs, only reaching statistical significance when comparing 40-keV images and CIs (*p* < 0.05) (Table 3).

**Fig. 1:**
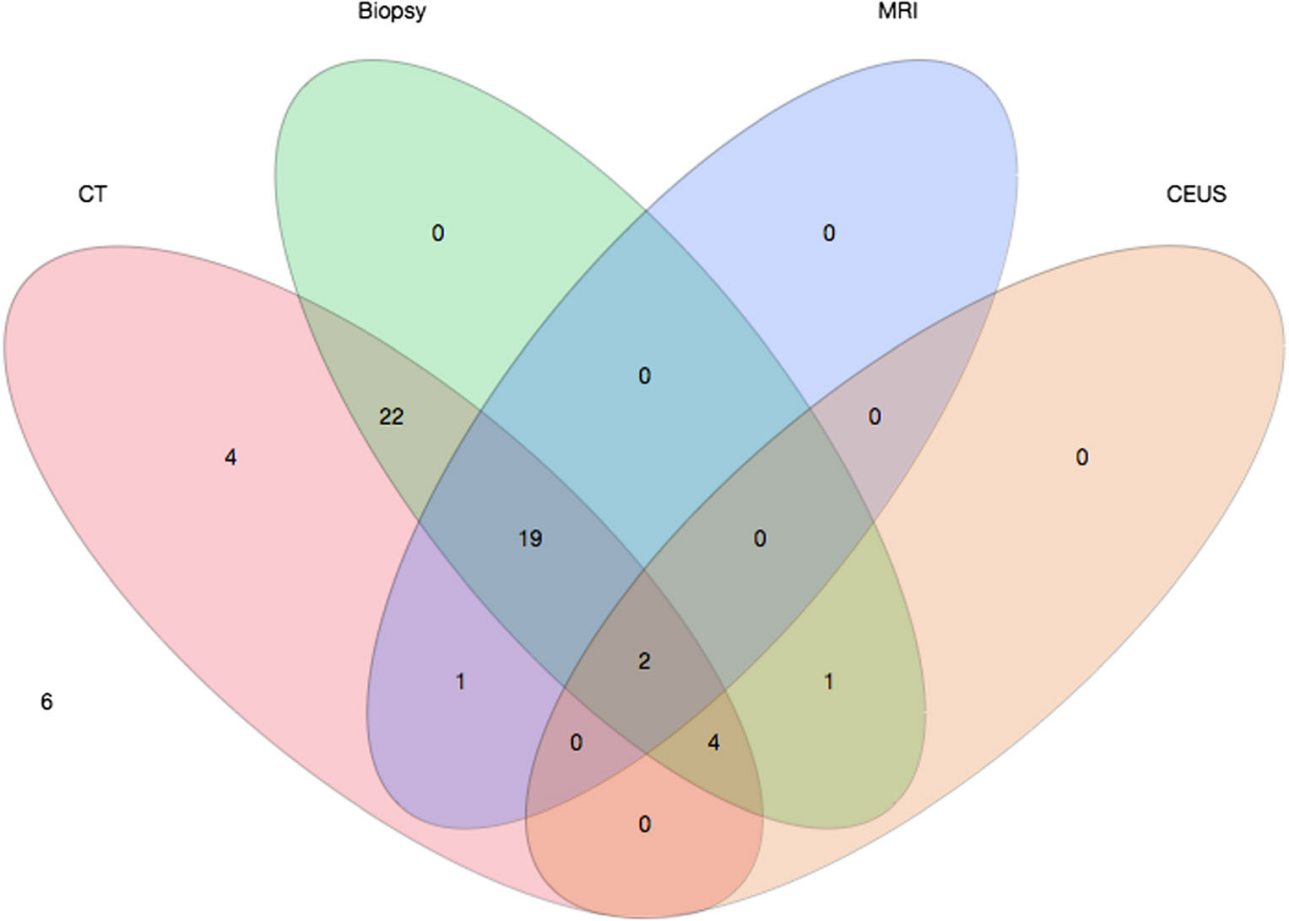
Venn diagram displaying the number of lesions with washout in any of the CT reconstructions diagnosed by any of the readers in comparison to the reference standards. Washout was diagnosed in all lesions with a positive reference standard, except for one lesion with a combination of a positive biopsy and washout in CEUS. Furthermore, both LiRADS 4 lesions with a capsule in MRI and 2 LiRADS 3 lesions without washout in MRI were diagnosed with washout in CT

Six arterially hyper-enhancing lesions exhibited no washout on any of the imaging modalities. In the remaining 53 lesions, washout was identified on CT images by at least one reader (Fig. 1). As mentioned above, washout was only (32.3%) or better (39.9%) seen on delayed-phase CT images in 72.2%, while it was better seen in portal-venous phase in 27.8%.

The diagnostic sensitivity to detect washout in LiRADS 4/5 lesions was highest in 40-keV VMIs (81.3% (73.8–86.5%)) followed by 60-keV (78.2% (70.9–84.1%)) and 80-keV VMIs (75.5% (68.0–81.8%)) and CIs (72.1% (64.4–78.7%)). Specificity was highest in CIs (96.3% (81.7–99.3%) followed by 60-keV (92.6% (76.6–97.9%), 80-keV (92.6% (76.6–97.9%) and 40-keV VMIs (88.9% (71.9–96.1%) (Table 3). Interestingly, 2 LIRADS 4 lesions with a capsule but no washout on MRI and 2 LIRADS 3 lesions without washout on MRI exhibited washout on SDCT (altogether 2 lesions on CIs, 2 lesions on 80-keV, 3 lesions on 60-keV and 4 lesions on 40-keV VMIs). On direct comparison, 40-keV VMIs were chosen to show best washout visibility in 88.3%, followed by 60-keV VMIs (5.1%), CIs (4.4%) and 80-keV VMIs (2.2%) (Table 3).

### Quantitative analysis

#### Image quality parameters

**Fig. 2:**
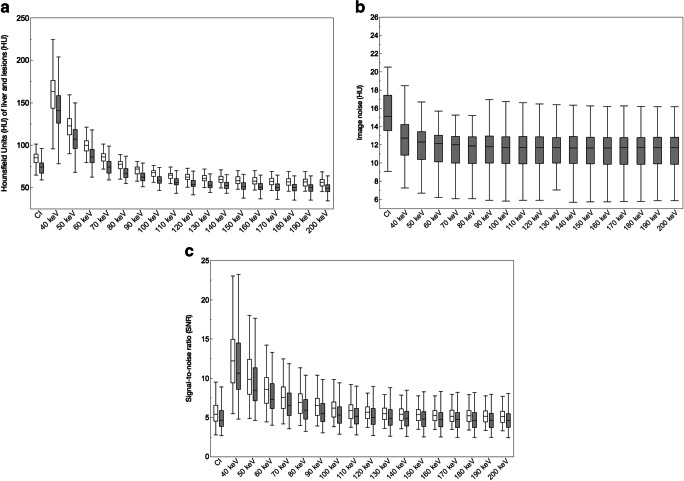
Illustration of mean Hounsfield units (HU) of liver parenchyma (empty boxplots) and lesions (filled boxplots) clearly reveals higher HU in low-kiloelectronvolt virtual monoenergetic images (VMIs) compared to conventional images (CIs) (**a**). Noise, as depicted by standard deviation (SD) of the autochthonous back musculature, differed in dependency of keV level in VMIs; however, it was found to be lower in any of the VMIs compared to CIs (**b**). This resulted in clear superiority of signal-to-noise ratio for liver (empty boxplots) and lesions (filled boxplots) in 40–90-keV images compared to CIs, respectively (**c**)

Lesions exhibited average HU of 75.4 ± 29.0 on CIs. For VMIs, a stepwise decrease of HU was found from low to high kiloelectronvolt. Compared to CIs and as expected, HU were significantly higher in VMIs with 40–60-keV levels and significantly lower in VMIs with 80–200 keV (*p* < 0.05; Fig. 2, Table 4). Image noise was highest in CIs (15.4 ± 3.0 HU) and significantly higher in 40-keV VMIs (12.8 ± 2.7 HU) compared to energy levels of 60-keV (11.7 ± 2.2 HU) and above (*p* < 0.05; Fig. 2, Table 4). SNRs of lesions and liver were significantly highest in 40-keV VMIs, respectively (*p* < 0.05). It decreased with increasing keV levels and was significantly higher in VMIs with 40–90-keV levels compared to CIs (*p* < 0.05; Fig. 2, Table 4).

**Table 4:** Quantitative results of image quality parameters

	CIs	40-keV VMIs	80-keV VMIs	120-keV VMIs	200-keV VMIs
Noise (SD_muscle_)	15.4 ± 3.0	12.8 ± 2.7	11.4 ± 2.1	11.3 ± 2.1	11.3 ± 2.1
Lesion					
Attenuation	74.8 ± 9.0	140.6 ± 25.4	67.9 ± 7.8	55.7 ± 6.8	50.7 ± 6.9
SNR	5.0 ± 1.6	11.8 ± 4.4	6.3 ± 1.9	5.3 ± 1.5	4.8 ± 1.4
Liver					
Attenuation	84.3 ± 8.9	160.9 ± 32.4	76.4 ± 6.6	62.3 ± 5.1	56.4 ± 5.5
SNR	5.6 ± 1.6	12.5 ± 4.1	7.0 ± 1.7	5.8 ± 1.4	5.3 ± 1.2

*CIs*, conventional images; *VMIs*, virtual monoenergetic images; *SD*, standard deviation; *SNR*, signal-to-noise ratio. Results are means ± standard deviation

#### Washout evaluation

The following results compare LiRADS 3 and 4/5 lesions where applicable.

On equivalent image reconstructions, CNR and quantitative washout values were significantly higher in LiRADS 4/5 compared to LiRADS 3 lesions (e.g. CNR in CIs 0.8 ± 0.5 vs. 0.3 ± 0.3, and washout in 40 keV 29.0 ± 19.1 vs. 15.1 ± 15.8 HU; *p* < 0.05).

**Fig. 3:**
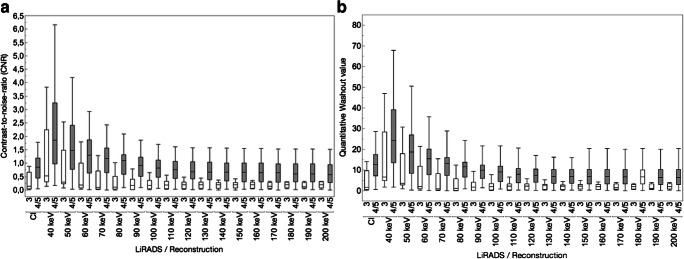
Illustrations clearly show a higher lesion-to-liver contrast-to-noise ratio (CNR) and quantitative washout values in LiRADS 4/5 (filled boxplots) compared to LiRADS 3 lesions (empty boxplots) (**a**, **b**)

In LiRADS 4/5 lesions, CNR and washout values decreased with increasing kiloelectronvolt levels. Both were significantly highest in 40-keV VMIs, while CNR was significantly higher in 40–70-keV levels compared to CIs and washout values were significantly higher in 40- and 50-keV levels compared to CIs (*p* < 0.05; Fig. 3, Table 5).

**Table 5:** Quantitative results of washout assessment

	CIs	40-keV VMIs	80-keV VMIs	120-keV VMIs	200-keV VMIs
CNR					
LiRADS 3	0.3 ± 0.3	1.2 ± 1.3	0.3 ± 0.4	0.2 ± 0.2	0.2 ± 0.1
LiRADS 4/5	0.8 ± 0.5	2.3 ± 1.6	1.0 ± 0.5	0.7 ± 0.4	0.6 ± 0.4
Washout					
LiRADS 3	4.5 ± 5.2	15.1 ± 15.8	3.3 ± 4.2	2.1 ± 1.8	2.0 ± 1.3
LiRADS 4/5	12.9 ± 6.9	29.0 ± 19.1	10.9 ± 5.6	8.2 ± 5.0	7.1 ± 5.2

*CIs*, conventional images; *VMIs*, virtual monoenergetic images; *CNR*, contrast-to-noise ratio; *LiRADS*, Liver Imaging Reporting and Data System. Results are means ± standard deviation

**Fig. 4:**
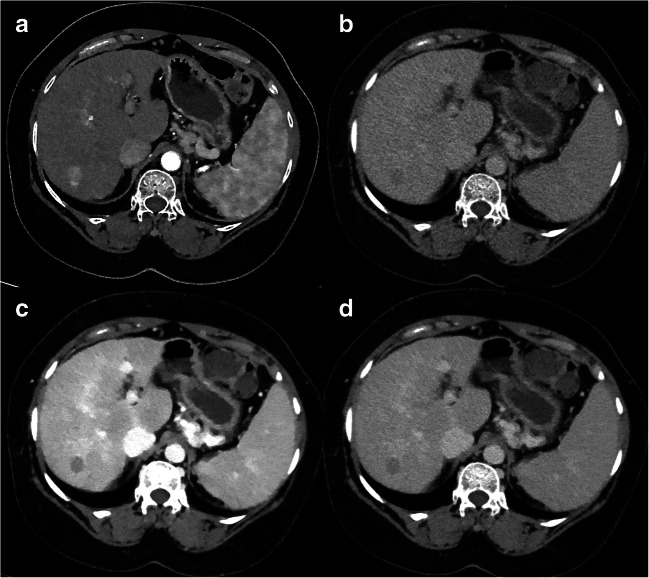
Axial CT images showing an arterially hyper-enhancing lesion in liver segment VII (**a**) with washout in delayed phase on CI (**b**), 40 keV (**c**) and 60 keV (**d**). However, washout and lesion-to-liver ratio were higher in 40-keV compared to 60-keV images and CIs, resulting in a better visibility

**Fig. 5:**
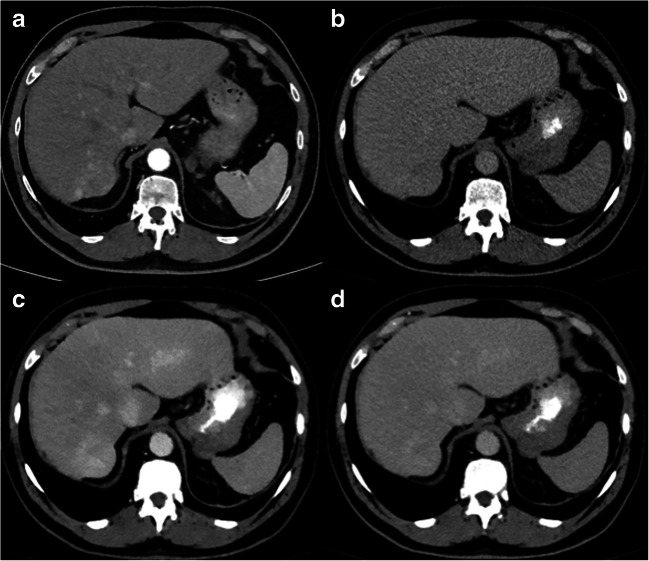
Axial CT images showing an arterially hyper-enhancing heterogeneous lesion in liver segment VII (**a**) with washout in delayed phase on CIs (**b**) and suggested on 60-keV images (**d**). However, the lesion partially exhibited hyperdense Hounsfield units on 40-keV images compared to the liver parenchyma (**c**). The lesion also showed washout in the reference MRI and was categorized as LiRADS 5, which was proved by biopsy

Image examples are shown in Figs. 4 and 5.

## Discussion

Our study confirms an improved washout assessment of arterially hyper-enhancing liver lesions for contrast-enhanced SDCT-derived low-kiloelectronvolt VMIs. Resulting from an increased soft tissue contrast, we observed best washout visibility, diagnostic sensitivity and confidence for 40-keV VMIs compared to all other tested reconstructions. With respect to washout evaluation in particular, overall ICC was excellent among the three readers.

So far, only few studies exist on the added value of DECT for the diagnostic workup of HCC [13, 14, 17]. Moreover, to our knowledge, there is only a single report on the added value of dual-energy CT for visual washout assessment in HCC [17]. In their study, Matsuda et al assessed washout visibility on virtual monoenergetic reconstructions acquired during equilibrium phase. Matsuda’s and our study differ in a number of aspects, in particular with respect to inclusion criteria (lesions > 1 cm fulfilling HCC criteria on conventional CT images vs. any arterially hyper-enhancing lesions > 1 cm), the injected contrast dose (adjusted to body surface area vs. fixed dose) and the timing of delayed-phase acquisition (100 s vs. 240 s after a trigger threshold of 100 and 150 HU, respectively) [17]. The longer delay chosen in our study is in line with protocol recommendations by the American College of Radiology, which advise a delay of 2–5 min [23]. On delayed-phase images, enhancement of liver parenchyma was well above the recommended 50 HU in all of our reconstructions. Despite the outlined differences, Matsuda et al reported similar results with significantly higher SNR, CNR and washout values in noise-reduced low mono-kiloelectronvolt images [17]. The improved image quality of SDCT-derived low-kiloelectronvolt images observed in our study is in accordance with earlier studies [10, 16, 24].

By also including lesions with no apparent signs of washout on conventional CT reconstructions in our study, we were able to demonstrate that low-kiloelectronvolt VMIs not only improve diagnostic confidence but also may increase the sensitivity for the detection of washout of arterially enhancing lesion on delayed-phase images. Depending on the reader, the number of lesions that were confidently identified to exhibit some degree of washout increased with the assessment of 40-keV VMIs by 10–35% compared to CIs. Moreover, all biopsy-proven lesions and 4 lesions with no signs of washout on MR imaging were identified to exhibit signs of washout on SDCT images. As a consequence, two LiRADS 3 and both LiRADS 4 lesions would have been upgraded to LiRADS 5 based on 40- and 60-keV images. In light of these results, it was surprising that one LiRADS 5 lesion with a heterogeneous APHE and obvious signs of washout on delayed-phase CIs and 80-keV images partially exhibited hyperdense HU on 40-keV images compared to the adjacent liver parenchyma hampering washout assessment in this case (Fig. 5). This case illustrates that VMIs should always be assessed in conjunction with CIs.

While a systematic comparison of the sensitivity of washout detection between portal-venous and delayed phases was not the main focus of our study, we observed a clear advantage for delayed-phase images with 72% of lesions exhibiting signs of washout only or better on delayed-phase images. This is in good agreement with several studies that have proven a clear benefit for delayed-phase images over portal-venous-phase images for the detection of lesion washout [25].

Other studies investigating HCC using DECT focussed on quantitative imaging parameters for decision-making [18, 26, 27]. For example, Pfeiffer et al proposed various imaging parameters using HU values and iodine concentration in and between arterial and portal-venous phase for the identification of HCC, while Laroia et al investigated iodine density of lesions in arterial phase for the assessment of HCC [18, 26].

Another study by Liu et al using conventional CT scanners introduced a percentage-ratio approach for HCC detection using the arterial, portal-venous and delayed phases [27]. While our quantitative measurements confirm an improved CNR between lesions with washout and adjacent liver parenchyma for low-kiloelectronvolt VMIs, subjective analysis of HCC remains the gold standard in clinical routine. This is especially true for heterogeneous lesions, in which only certain areas may exhibit signs of washout.

Besides the retrospective study design, several limitations need to be addressed. First, the study only includes a limited number of patients and lesions. Second, histological confirmation and grading was not available for all lesions, especially no large population of dysplastic nodules of various grading or early HCCs were included in this first study using SDCT. Also MRI was not available in all cases impeding a direct comparison of diagnostic performance between both modalities. As a consequence, estimates of diagnostic accuracy need to be interpreted with caution. In particular, the reduced specificity of 40- and 60-keV images for washout detection should not be interpreted as an increased rate of false positives, but is more likely to be explained by the limited sensitivity of our reference imaging modalities. Fourth, the impact of different-sized ROIs on our results was not assessed. Furthermore, it must be emphasized that conventional CT images remain the gold standard, while VMIs may be beneficial to be additionally reviewed in uncertain situations. Last, we quantitatively and qualitatively assessed washout of known arterially hyper-enhancing lesions in this proof of concept study; however, based on these results, a further large-scale study to investigate the value of SDCT in comparison to CT, MRI and CEUS for non-invasive detection and characterization of liver lesions in high-risk patients is desired. Its exact impact on the diagnostic accuracy in comparison to the other modalities remains to be determined.

In conclusion, virtual monoenergetic images obtained with spectral detector CT using low-kiloelectronvolt images may improve the assessment of washout in arterially hyper-enhancing liver lesions. Hence, these reconstructions seem beneficial in uncertain situations to improve confidence of washout evaluation and visibility in delayed-phase images.

## Electronic supplementary material

ESM 1 (DOCX 138 kb)

## Abbreviations

CE: Contrast-enhanced
CIs: Conventional images
DECT: Dual-energy CT
HCC: Hepatocellular carcinoma
keV: Kiloelectronvolt
SDCT: Spectral detector CT
VMIs: Virtual monoenergetic images

## References

[1] Marrero JA, Kulik LM, Sirlin CB (2018). Diagnosis, staging, and management of hepatocellular carcinoma: 2018 Practice Guidance by the American Association for the Study of Liver Diseases. Hepatology.

[2] Galle PR, Forner A, Llovet JM (2018). EASL Clinical Practice Guidelines: Management of hepatocellular carcinoma. J Hepatol.

[3] Li J, Wang J, Lei L (2019). The diagnostic performance of gadoxetic acid disodium-enhanced magnetic resonance imaging and contrast-enhanced multi-detector computed tomography in detecting hepatocellular carcinoma: a meta-analysis of eight prospective studies. Eur Radiol.

[4] Hanna RF, Miloushev VZ, Tang A (2016). Comparative 13-year meta-analysis of the sensitivity and positive predictive value of ultrasound, CT, and MRI for detecting hepatocellular carcinoma. Abdom Radiol (NY).

[5] American College of Radiology: Liver Imaging Reporting and Data System (2018) CT/MRI LI-RADS® v2018. https://www.acr.org/Clinical-Resources/Reporting-and-Data-Systems/LI-RADS/CT-MRI-LI-RADS-v2018. Accessed 20 Jan 2019

[6] Dietrich CF, Fetzer D, Jang H-J et al (2017) CEUS LI-RADS ® v2017 CORE. In: Am. Coll. Radiol. https://www.acr.org/Clinical-Resources/Reporting-and-Data-Systems/LI-RADS/CEUS-LI-RADS-v2017. Accessed 8 Oct 2019

[7] Navin PJ, Venkatesh SK (2019). Hepatocellular carcinoma: state of the art imaging and recent advances. J Clin Transl Hepatol.

[8] Alvarez RE, Macovski A (1976). Energy-selective reconstructions in X-ray computerized tomography. Phys Med Biol.

[9] Neuhaus V, Abdullayev N, Große Hokamp N (2017). Improvement of image quality in unenhanced dual-layer CT of the head using virtual monoenergetic images compared with polyenergetic single-energy CT. Invest Radiol.

[10] Große Hokamp N, Höink AJ, Doerner J (2018). Assessment of arterially hyper-enhancing liver lesions using virtual monoenergetic images from spectral detector CT: phantom and patient experience. Abdom Radiol (NY).

[11] McCollough CH, Leng S, Yu L, Fletcher JG (2015). Dual- and multi-energy CT: principles, technical approaches, and clinical applications. Radiology.

[12] Lennartz S, Laukamp KR, Neuhaus V (2018). Dual-layer detector CT of the head: initial experience in visualization of intracranial hemorrhage and hypodense brain lesions using virtual monoenergetic images. Eur J Radiol.

[13] Shuman WP, Green DE, Busey JM (2014). Dual-energy liver CT: effect of monochromatic imaging on lesion detection, conspicuity, and contrast-to-noise ratio of hypervascular lesions on late arterial phase. AJR Am J Roentgenol.

[14] Lv P, Lin XZ, Chen K, Gao J (2012). Spectral CT in patients with small HCC: investigation of image quality and diagnostic accuracy. Eur Radiol.

[15] Husarik DB, Gordic S, Desbiolles L (2015). Advanced virtual monoenergetic computed tomography of hyperattenuating and hypoattenuating liver lesions: ex-vivo and patient experience in various body sizes. Invest Radiol.

[16] Große Hokamp N, Obmann VC, Kessner R (2018). Improved visualization of hypodense liver lesions in virtual monoenergetic images from spectral detector CT: proof of concept in a 3D-printed phantom and evaluation in 74 patients. Eur J Radiol.

[17] Matsuda M, Tsuda T, Kido T (2018). Dual-energy computed tomography in patients with small hepatocellular carcinoma: utility of noise-reduced monoenergetic images for the evaluation of washout and image quality in the equilibrium phase. J Comput Assist Tomogr.

[18] Laroia ST, Bhadoria A, Venigalla Y (2016). Role of dual energy spectral computed tomography in characterization of hepatocellular carcinoma: initial experience from a tertiary liver care institute. Eur J Radiol Open.

[19] Hokamp NG, Maintz D, Shapira N (2020). Technical background of a novel detector-based approach to dual-energy computed tomography. Diagn Interv Radiol.

[20] Zopfs D, Laukamp KR, Pinto dos Santos D (2019). Low-keV virtual monoenergetic imaging reconstructions of excretory phase spectral dual-energy CT in patients with urothelial carcinoma: a feasibility study. Eur J Radiol.

[21] Gamer M, Lemon J, Fellows I, Singh P (2012) Package irr: various coefficients of interrater reliability and agreement. Available via https://cran.r-project.org/web/packages/irr/irr.pdf. Accessed 25 Mar 2019

[22] Cohen J (1960). A coefficient of agreement for nominal scales. Educ Psychol Meas.

[23] Kambadakone A, Chandarana H, Chernyak V et al (2018) LI-RADS ® Technique. Am Coll Radiol. Available via https://www.acr.org/-/media/ACR/Files/Clinical-Resources/LIRADS/Chapter-12-Technique.pdf?la=en&hash=3B774BD8A6D0A6ACBD62B2330705FD14. Accessed 12 Mar 2019

[24] Große Hokamp N, Obmann VC, Kessner R (2018). Virtual monoenergetic images for diagnostic assessment of hypodense lesions within the liver: semiautomatic estimation of window settings using linear models. J Comput Assist Tomogr.

[25] Monzawa S, Ichikawa T, Nakajima H (2007). Dynamic CT for detecting small hepatocellular carcinoma: usefulness of delayed phase imaging. AJR Am J Roentgenol.

[26] Pfeiffer D, Parakh A, Patino M (2018). Iodine material density images in dual-energy CT: quantification of contrast uptake and washout in HCC. Abdom Radiol (NY).

[27] Liu YI, Shin LK, Jeffrey RB, Kamaya A (2013). Quantitatively defining washout in hepatocellular carcinoma. AJR Am J Roentgenol.

