# Supplementary figures and images for: Virtual monoenergetic images from spectral detector computed tomography facilitate washout assessment in arterially hyper-enhancing liver lesions
Source: Eur Radiol. 2020 Nov 12;31(5):3468–77. doi: 10.1007/s00330-020-07379-3 (PMC8043945; doi:10.1007/s00330-020-07379-3)

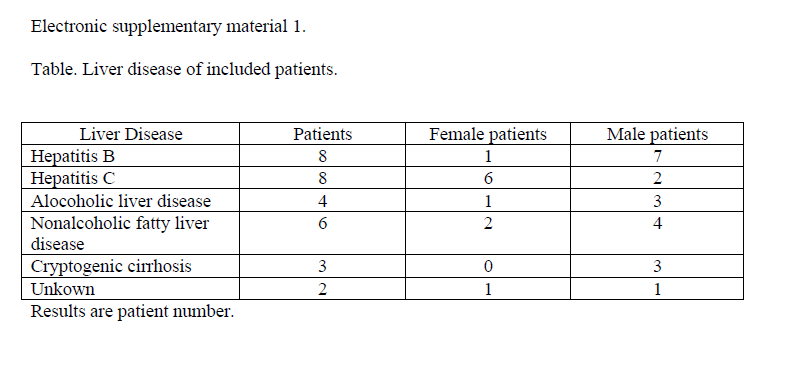


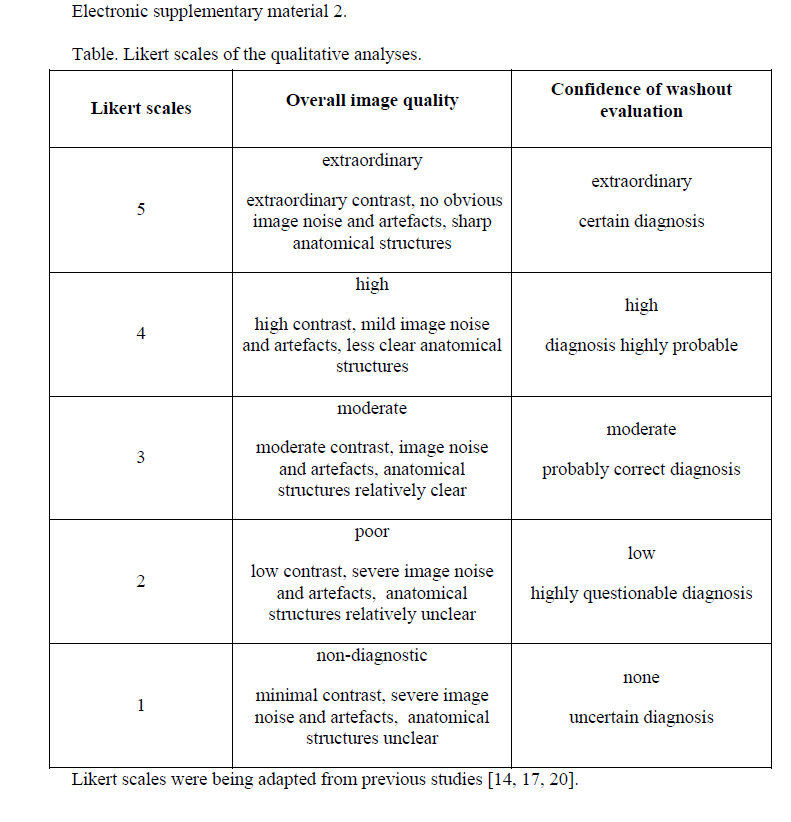

Supplement: Supplementary file 1 — (DOCX 138 kb) [file 330_2020_7379_MOESM1_ESM.docx]
